# Immunological alteration and changes of gut microbiota after dextran sulfate sodium (DSS) administration in mice

**DOI:** 10.1007/s10238-013-0270-5

**Published:** 2014-01-11

**Authors:** Å. Håkansson, N. Tormo-Badia, A. Baridi, J. Xu, G. Molin, M.-L. Hagslätt, C. Karlsson, B. Jeppsson, C. M. Cilio, S. Ahrné

**Affiliations:** 1 Food Hygiene, Division of Applied Nutrition and Food Chemistry, Lund University, Lund, Sweden; 2 Cellular Autoimmunity Unit, Department of Clinical Sciences, Malmö University Hospital, Lund University, Malmö, Sweden; 3 Department of Surgery, Malmö University Hospital, Lund University, Malmö, Sweden

**Keywords:** Inflammation, T-RFLP, FACS, Immunological reactions, Microbial composition

## Abstract

Ulcerative colitis (UC) is characterized by chronic inflammation of the colonic mucosa. Administration of dextran sulfate sodium (DSS) to animals is a frequently used model to mimic human colitis. Deregulation of the immune response to the enteric microflora or pathogens as well as increased intestinal permeability have been proposed as disease-driving mechanisms. To enlarge the understanding of the pathogenesis, we have studied the effect of DSS on the immune system and gut microbiota in mice. Intestinal inflammation was verified through histological evaluation and myeloperoxidase activity. Immunological changes were assessed by flow cytometry in spleen, Peyer′s patches and mesenteric lymph nodes and through multiplex cytokine profiling. In addition, quantification of the total amount of bacteria on colonic mucosa as well as the total amount of lactobacilli, *Akkermansia*, *Desulfovibrio* and *Enterobacteriaceae* was performed by the use of quantitative PCR. Diversity and community structure were analysed by terminal restriction fragment length polymorphism (T-RFLP) patterns, and principal component analysis was utilized on immunological and T-RFLP patterns. DSS-induced colitis show clinical and histological similarities to UC. The composition of the colonic microflora was profoundly changed and correlated with several alterations of the immune system. The results demonstrate a relationship between multiple immunological changes and alterations of the gut microbiota after DSS administration. These data highlight and improve the definition of the immunological basis of the disease and suggest a role for dysregulation of the gut microbiota in the pathogenesis of colitis.

## Introduction

The immune system is pivotal in mediating the interactions between the host and the intestinal bacteria, while the composition of the bacterial flora in the gut strongly influences the outcome of the immune response [1]. Some microorganisms seem to promote immune regulation, whereas potential pathogenic bacteria under certain circumstances may trigger intestinal inflammation in susceptible hosts [1].

Ulcerative colitis (UC) is an inflammatory disease of the rectal and colonic mucosa, and clinical symptoms include weight loss, diarrhoea accompanied by rectal bleeding and abdominal pain [2]. Affected areas indicate transmural inflammation characterized by lymphoid hyperplasia, submucosal oedema, ulcerative lesions and fibrosis [3]. During clinical relapses with acute inflammation, the mucosal lining of the intestine displays a characteristic inflammatory infiltrate of mast cells, lymphocytes, macrophages and activated neutrophils [4]. In turn, intestinal inflammation may enhance permeability increasing the risk of bacterial translocation and endotoxemia [5]. The interaction between endotoxin and monocytes/macrophages through toll-like receptors induces a variety of intracellular signalling cascades, finally leading cytokine production [6].

The dextran sulfate sodium (DSS) model, originally reported by Okayasu et al., has been extensively used to investigate the role of various leucocytes during severe colitis [7]. The advantage of the model is its resemblance to UC, exhibiting several morphological and pathophysiological features such as superficial ulceration, mucosal damage, production of cytokines and other inflammatory mediators and leucocyte infiltration [7–9]. The outcome of DSS-induced colitis is dependent on the genetic background of the animals, and the inflammatory response is primarily affecting the distal colon, although in some rodent strains, the inflammation can even be found in proximal colon, caecum and the distal small bowel [10, 11].

Although there is overwhelming support for the hypothesis that intestinal inflammation is triggered by enteric bacteria, the specific antigens that drive the immune response have not yet been identified. Understanding how the immune system is regulated and responds to variations in the composition of the intestinal microflora may require whole-system approaches, since the study of single immunological parameters is inadequate in unravel the complexity of the immune system. In the present study, we attempted to clarify the associations of the colonic microflora with differences in immunological profiles in DSS-induced colitis in mice. Evaluations of immune functions were assessed through flow cytometry of lymphocyte subpopulations, macrophages and dendritic cells in spleen, Peyer′s patches and mesenteric lymph nodes, as well as through multiplex cytokine profiling of serum samples. The quantification of the total amount of bacteria as gene copy numbers on colonic mucosal samples as well as gene copy numbers of lactobacilli, *Akkermansia*, *Desulfovibrio* and *Enterobacteriaceae* was analysed by qPCR. Assessment of the diversity of the colonic mucosal microbiota and community structure among individuals was established by use of terminal restriction fragment length polymorphism (T-RFLP) patterns. Principal component analysis was performed on immunological parameters and T-RFLP patterns.

Intestinal inflammation was verified through histological evaluation and myeloperoxidase (MPO) activity.

## Materials and methods

### Animals

Wild-type female C57BL/6 mice (Charles Rivers Laboratories International, Inc., Germany) were kept under standardized conditions in the animal facility and were allowed unrestricted access to standard chow and tap water. Acclimatization for at least 7 days to the laboratory conditions before experimental inclusion was performed. All experiments were performed in compliance with the relevant Swedish and institutional laws and guidelines.

### Induction of colitis and study design

Twenty mice were randomly allocated to one of two experimental groups (*n* = 10), the control group with untreated animals (NC group) and the group receiving DSS (DSS group). Acute colitis was induced by feeding the animals ad libitum with 4 % (wt/vol) DSS (molecular weight 40 kDa; ICN Biochemicals, Aurora, OH) dissolved in drinking water, for 7 days. Drinking volumes were recorded every 48 h for each animal, and the DSS load was calculated as:

Total drinking water (ml) × (DSS (g)/100 ml).

On day 7, post-induction of colitis, the animals were killed by cervical spine dislocation and arterial blood was withdrawn for cytokine assay. Under an aseptic technique, a laparotomy was performed through a midline incision, and all detectable mesenteric lymph nodes, the left lobe of the liver, spleen, small intestine, caecum and the entire colorectum from the colocaecal junction to the anal verge were excised. Peyer′s patches of whole small bowel (proximal, middle and distal) were isolated and assigned for flow cytometry analysis along with mesenteric lymph nodes and spleen. The length of colon as well as wet weights of caecum, colon and spleen was documented. When measuring length, all intestinal segments were vertically suspended with a weight of 1.5 g to provide uniform tension. The luminal content of caecum and colon was gently removed, and caecal tissue was rinsed with isotonic saline before placed in an 80 °C oven for 48 h, then reweight after 24 and 48 h, and the wet-to-dry weight ratio was determined as a measure of oedema [12]. Intestinal samples from small intestine and colon were saved for histopathological and microbial (only colon) evaluation as well as for MPO activity. The samples were immediately placed in 4 % formaldehyde (histopathological evaluation) or frozen in liquid nitrogen for later determination (microbial evaluation and MPO). Samples from the small intestine were taken from duodenum to jejunum, and samples from colon–rectum were taken from the posterior rectum to the mid-part.

### Clinical scoring of colitis

Over the 7-day study protocol, animals were monitored daily on the basis of weight loss, stool consistency and rectal bleeding to obtain a clinical index of disease activity (DAI). The scoring system has been validated and shown to correlate histologically with pathological findings [8, 13]. The DAI was scored on a scale of 0–4 for each clinical parameter and then averaged for each animal. Weight loss, stool and bleeding scores were defined by modified scoring limits [14].

### Histology

Specimens from the distal part of colon and liver were evaluated by a blinded scientist using light microscopy. One-centimetre-long specimens from the most distal part of the colon, and the left lobe of the liver, were each fixed in neutral buffered formalin, followed by standard procedure for paraffin embedding. Serial sections were cut for each organ and stained with haematoxylin-eosin staining. The evaluation of histopathological changes in the colon was done according to Cooper et al. [8].

### MPO activity

MPO activity, used for quantification of neutrophil infiltration, was estimated in the whole colonic tissue, containing mucosa and muscle layers [15]. Specimens of distal colon were collected and weighed prior to storage at −70 °C until time of assay. The assay procedure was done in accordance with Osman et al. [16]. The activity was expressed as units/gram of wet weight tissue.

### DNA extraction

DNA extraction from colonic mucosal samples was done by using BioRobot EZ1 and DNA Tissue Kit (Qiagen) as described elsewhere [17].

### Terminal restriction fragment length polymorphism (T-RFLP) analysis

#### PCR amplification for T-RFLP analysis

The 16S rRNA genes were amplified using primer FAM-ENV1 (5′-AGA GTT TGA TII TGG CTC AG-3′) and ENV2 (5′-CGG ITA CCT TGT TAC GAC TT-3′) [18]. The forward primer ENV1 was fluorescently labelled with FAM (Applied biosystems, Foster city, CA, USA) at 5′ end. The PCR mixture, in a total volume of 25 μl, contained 0.4 μM of primer FAM-ENV1 and 0.2 μM of primer ENV2, 2.5 μl of 10 × PCR buffer (500 mM Tris–HCl, 100 mM KCl, 50 mM (NH_4_)_2_SO_4_, 20 mM MgCl_2_, pH 8.3), 0.2 mM of each deoxyribonucleotide triphosphate, 2.5 U of FastStart Taq DNA polymerase (Roche Diagnostics, Mannheim, Germany) and 2 μl of template DNA. The PCR was performed in an Eppendorf MasterCycler (Eppendorf, Hamburg, Germany) using the following programme: 95 °C for 3 min, 94 °C for 3 min, followed by 30 cycles of 94 °C for 1 min, 50 °C for 45 s and 72 °C for 2 min. Finally, an additional extension at 72 °C for 7 min was done. Triplicate reactions were carried out for each sample, and a negative control was included in all the PCR runs. After the amplification, the PCR products were verified by Agarose Gel Electrophoresis. PCR products of each sample were then pooled and purified by MinElute PCR Purification Kit (Qiagen, Hilden, Germany) according to the manufacturer’s protocol. DNA was eluted in 30 μl of sterile water, and DNA concentration was measured by Nanodrop ND-1000 (Saveen Werner, Limhamn, Sweden).

#### T-RFLP analysis

Two hundred nanograms of the purified PCR products was digested separately with 10 U of the restriction endonucleases *Msp*I (Fermentas Life Science, Burlington, Canada) in a total volume of 10 μl for 5 h at 37 °C. The enzyme was then inactivated by heating at 65 °C for 20 min. After digestion, aliquots of the products were diluted 5 times with sterile water in a sterile 96-well plate (Becton–Dickinson, Franklin Lakes, NJ, USA). Samples were then sent to DNA-lab Malmö University Hospital (UMAS) for the T-RFLP analysis. Analysis was performed on a 3130xl Genetic Analyser (Applied Biosystems), and in all samples, a DNA size marker GeneScan™ LIZ 600 (Applied Biosystem) was included. Fragment sizes, peak height and peak area were analysed with Genemapper^®^ software version 4.0 (Applied Biosystems). Local Southern method was chosen for size calling, and the size range was set from 40 to 580 bp. The peak amplitude thresholds were set to 50 relative fluorescence units (rfu) for samples and 10 rfu for standards. The total peak area for each sample was calculated by summarizing the area for all peaks in a sample. The relative peak area of each peak was expressed as percentage of the total area.

### Quantitative real-time polymerase chain reaction (qPCR)

Standards for the qPCR that were used to quantify the *Lactobacillus* and *Enterobacteriaceae* were prepared by cloning of the corresponding partial 16S rDNA fragments. The fragment of interest was amplified from *Lactobacillus* *plantarum* 299v and *Escherichia* *coli* CCUG 29300^T^, respectively, using primers listed in Table 1 [19–21].

**Table 1:** Primers used in qPCR to amplify four target regions

Name	Sequence (5′–3′)	Target group	Amplicon size (bp)	Template DNA	Annealing term (°C)	References
Lact-F	AGCAGTAGGGA ATCTTCCA (19)	*Lactobacillus*	341	*L. plantarum* 299v	61	Walter et al. [19]
Lact-R	CACCGCTACAC ATGGAG (17)					Heilig et al. [20]
Uni331-F	TCCTACGGGAG GCAGCAGT (19)	Total bacteria	466	*L. plantarum* 299v	58	Nadkarni et al. [22]
Uni797-R	GGACTACCAGG GTATCTAATCCTGTT (26)					
Ecol457-F	CATTGACGTTAC CCGCAGAAGAAGC (25)	*Enterobacteriaceae*	195	*E.coli*	60	Bartosch et al. [21].
Ecol652-R	CTCTACGAGACT CAAGCTTGC (21)					
AMI-F	CAGCACGTGAA GGTGGGGGACC (20)	*Akkermansia*	327	*A. municiphila*	60	Collado et al. [23]
AM2-R	CCTTGCGGTTGG CTTCAGAT (20)					
DSV691-F	CCGTAGATATCT GGAGGAACATCAG	*Desulfovibrio* sp.	135	*D. desulfuricans* subsp. *desulfuricans*	62	Fite et al. [24]
DSV826-R	ACATCTAGCATC CATCGTTTACAGC					

For total bacteria, another set of primers was used with *L. plantarum* 299v DNA as template (Table 1) [22]. For the preparation of a standard for *Akkermansia muciniphila,* a clone obtained from mice was used [23]. Specific primers developed for quantitating intestinal *Desulfovibrio* were also applied (Table 1) [24]. After amplification (Table 1), the PCR products were purified using a Wizard^®^ SV Gel and PCR Clean-Up System (Promega, Madison, USA) and cloned by using pGEM-T vector system (Promega, Madison, USA) into *E. coli* JM109.

Clones with correct inserts were cultured in LB-broth with ampicillin at 37 °C overnight. The plasmid DNA was extracted by using QIAprep^®^ Miniprep kit (Qiagen). The concentrations of the plasmid DNA were measured by Nanodrop ND-1000 (Saveen Werner, Limhamn, Sweden). For preparation of the standards, tenfold dilution series were made of the extracted plasmid DNA in TE buffer (10 mM Tris, 1 mM EDTA, pH 8.0) supplemented with 0.1 μg/μl Herring sperm DNA (VWR International, West Chester, PA, USA) and the copy numbers of each standard were calculated.

Quantitative PCR (qPCR) was performed in a Mastercycler^®^ ep realplex 1.5 real-time PCR system (Eppendorf) separately for the different groups of bacteria. The qPCR consisted of 10 μl of 2X Platinum^®^SYBR^®^ Green qPCR SuperMix-UDG (Invitrogen A/S, Taastrup, Denmark), 0.5 μM each of the forward and the reverse primer (Table 1) and 2 μl of template DNA in a final volume of 20 μl. Triplicate of standards and samples as well as triplicate negative controls was prepared in a sterile 96-well polypropylene microplate (Eppendorf). The qPCR was run under the following conditions. Initially, the temperature were set to 50 °C for 2 min, followed by 95 °C for 2 min; 40 cycles were then run with the following parameters: 95 °C for 15 s, primer annealing for 30 s and 72 °C for 30 s (Table 1). For amplification of the total bacteria, the elongation time was set for 45 s at 72 °C. Finally, a melting curve analysis was performed by a temperature gradient from 60 to 95 °C for 20 min and a final denaturation at 95 °C for 15 s. For data analysis, CalQplex algorithm and automatic baseline with drift correction were chosen for all the quantifications.

### Flow cytometry

Cells from spleen, mesenteric lymph nodes and Peyer′s patches were harvested using cell strainers (Becton, Dickinson and Company, USA). Red blood cells in spleen were lysed with ddH_2_O for 13 s in RT. Cells from all tissues were washed twice with Hanks-BSS (Gibco, Invitrogen, Paisley, UK) at 1,300 rpm for 10 min. The cells were counted, and approximately 1.5 × 10^6^ cells were plated for each staining in a 96-well round-bottom plate. After washing the plated cells with 200 μL staining buffer (1× PBS (AppliChem GmbH, Darmstadt, Germany), 0.1 % NaAz (Scharlau Chemie S.A., Sentmenat, Spain), 2 % foetal bovine serum (VWR International, Sweden)) at 1,800 rpm for 1 min, the cells were stained in 25 μL antibody solution for surface markers (CD11b, CD11c, CD8 (BD Pharmingen™, USA); CD3, CD4, CD25, CD49b, B220, CCR9 (BioLegend, San Diego, USA); CD69 (eBioscience, Inc., San Diego, USA)) for 30 min at 4 °C in dark. After staining, cells were washed twice with 175 μL FACS buffer (1× PBS (Applichem, Germany), 0.1 % NaAz (Scharlau Chemie S.A., Sentmenat, Spain), 2 % foetal bovine serum (VWR International, Sweden)) at 1,800 rpm for 1 min and re-suspended in 200 μL FACS buffer supplemented with 2 % formaldehyde (Apoteket, Sweden). For intranuclear (FoxP3 (eBioscience, Inc., San Diego, USA) and intracellular (CTLA-4 (BioLegend, San Diego, USA) markers, cells were fixed and permeabilized according to manufacturer’s protocol and then re-suspended in 200 μL FACS buffer. Stained cells were stored at 4 °C until FACS analysis performed next morning. The following antibodies combinations were used:

CD3/B220/CD8/CD4; CD3/CD25/CD8/CD4; CD4/CD25/FoxP3/CD69; CD4/CTLA-4/CD25/FoxP3; CD4/CD69/CCR9/CD8; CD3/CD49b/CD8/CD4; dump channel^*^ (CD11c/CD11b); dump channel^*^ (CD11c/TLR 4); dump channel^*^ (CD11c/CD11b/TLR 4).

^*^dump channel: CD3, B220, CD4, CD8

### Flow cytometry analysis

The FACS analysis was performed on FACS Calibur (Becton, Dickinson and Company, USA), and 30,000 lymphocytes in live gate were acquired for analysis. The data were analysed in FlowJo software (Treestar, Inc., Ashland, USA).

### Multiplex serum cytokine/chemokine profiling

Blood samples were allowed to clot at room temperature for 2 h before centrifugation (3,000 g, 4 °C, 10 min), and the serum was collected and stored at −80 °C until analysed. For quantitative analysis of cytokines/chemokines (interleukin (IL)-1β, IL-2, IL-4, IL-5, IL-6, IL-10, IL-12, IL-17, TNF-α, IFN-γ and KC), serum samples were thawed and run in duplicate using MILLIPLEX™ micro-beads array system following the manufacturer’s recommended protocols. The results were read by use of Luminex 100 v2.3, and for evaluation of the results, Milliplex™ Analyst v3.4 (Vigenetech) was used.Values below range were set at the value of detection limit.

### Statistical analysis

Feed intake, body weight change, caecal weight, colon length and weight, spleen weight, DAI scores, MPO activity, cytokines/chemokines and qPCR results (Fig. 6; Tables 2, 4) were presented as medians with 25 and 75 percentiles. The statistics were conducted in SigmaStat^®^ version 3.0 (SPSS Inc., Chicago, Ill., USA). The differences between experimental groups were assessed by a Mann–Whitney rank sum test. The correlation between expectations of benefit was ascertained using Spearman’s rank-order correlation. Calculation of the incidence of *Enterobacteriaceae* growth and the incidence of T-RFs were conducted in QuickStat version 2.6 and were evaluated by the Fisher’s exact test. To assess the difference in percent gated cells between DSS and NC, the Mann–Whitney U test was used. In Figs. 2, 3 and 4, outliers are shown as ° and extreme values are shown as *. The statistical tests were performed in SPSS 18.0 for Windows (SPSS Inc., Chicago, IL, USA). *P* values below 0.05 were regarded as statistically significant.

**Table 2:** Feed intake, body weight change, DAI, caecal wet/dry weight, colon length and colon wet weight in the normal control group (NC) and in the group receiving DSS (DSS)

	NC	DSS
Total feed intake (g)	23.0 (21.1 to 23.6) (n = 9)	16.5 (15.3 to 17.3) (n = 8)***
Body weight change (g)	0.3 (−0.5 to 1.0) (n = 10)	−4.0(−5.5 to (−3.0)) (n = 10)***
Body weight change (g/g feed)	0.02 (−0.03 to 0.04) (n = 9)	−0.3(−0.4 to (−0.2)) (n = 8)***
Disease activity index (day 7)	0.0 (0.0 to 0.3) (n = 10)	2.8 (2.3 to 3.0)(n = 10)***
Caecal wet/dry weight (g)	3.7 (3.3 to 4.5) (n = 10)	5.8 (4.7 to 6.0) (n = 10)***
Colon length (cm)	7.9 (7.5 to 8.2) (n = 10)	6.3 (5.9 to 7.0) (n = 10)***
Colon wet weight (g)	0.18 (0.15 to 0.20) (n = 9)	0.29 (0.24 to 0.30) (n = 9)***

Data are expressed as median values, range is presented within brackets. Asterisks indicate statistically significant difference from NC group: ***p < 0.001

## Results

### Feed intake and body weight changes

Feed intake and body weight change for the two groups are summarized in Table 2. Colitis induction resulted in a decrease in the consumption of feed (*P* < 0.001). All animals in the DSS group lost weight, and the body weight change (g) (*P* < 0.001) as well as body weight change/g feed (g/g feed) (*P* < 0.001) was significantly lower compared with the NC group.

### DSS consumption and DAI

No significant difference in DSS consumption was found between the individual animals (data not shown), and the median value of the total consumption in the group was 2.28 (2.08–2.58) g DSS. Oral administration of DSS-induced acute colitis in C57BL/6 mice, and on the 7th day, a significant difference was found in DAI between the NC group [0.0 (0.0–0.3)] and the DSS group [2.8 (2.3–3.0)] (*P* < 0.001) (Table 2).

### Colonic length and tissue weights

The DSS group got shorter colons (*P* < 0.001) (Table 2) and the thickness of the colonic tissue was increased, as reflected by the significantly increased colonic weight (*P* < 0.001) (Table 2). Wet-to-dry weight ratios of caecum were significantly increased in the DSS group, indicating oedema (Table 2), but no difference in spleen weight was observed between the groups [NC; 0.085 g (0.075–0.087), DSS; 0.081 g (0.076–0.091)].

### Histological evaluation

#### Colon

**Fig. 1:**
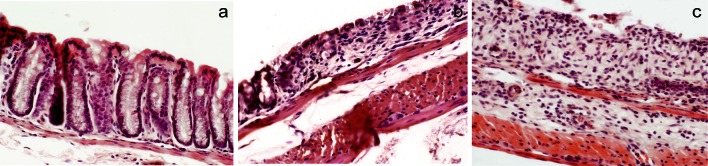
Histological analysis of intestinal mucosa. **a** Normal crypt architecture and the absence of inflammation in colonic mucosa (NC group). **b** Grade 2 lesion of colonic mucosa (DSS group). **c** Grade 3 lesion of colonic mucosa (DSS group)

Five preparations from each group were evaluated. No histopathological changes were found in the NC group (Fig. 1a). In the DSS group, three preparations showed grade 1 lesions and two showed grade 2 and grade 3–4 lesions, respectively (Fig. 1b, c).

#### Small intestine and liver

Histological evaluation of small intestinal samples did not reveal any abnormalities like crypt distortion or inflammatory infiltration in the NC group or the DSS group.

No histopathological changes related to steatosis or inflammatory infiltration could be observed in any of the liver preparations of the groups (data not shown)

### MPO activity

The MPO activity was significantly elevated in colonic samples from the DSS group [2.53 U/g tissue (1.69–3.50), NC group 0.16 U/g tissue (0.10–0.30)], (*P* < 0.001), whereas no difference between the groups was found in small intestinal samples (NC group; 0.55 U/g tissue (0.42–0.62), DSS group; 0.43 U/g tissue (0.3–0.74)).

### Immunological changes after DSS administration

**Fig. 2:**
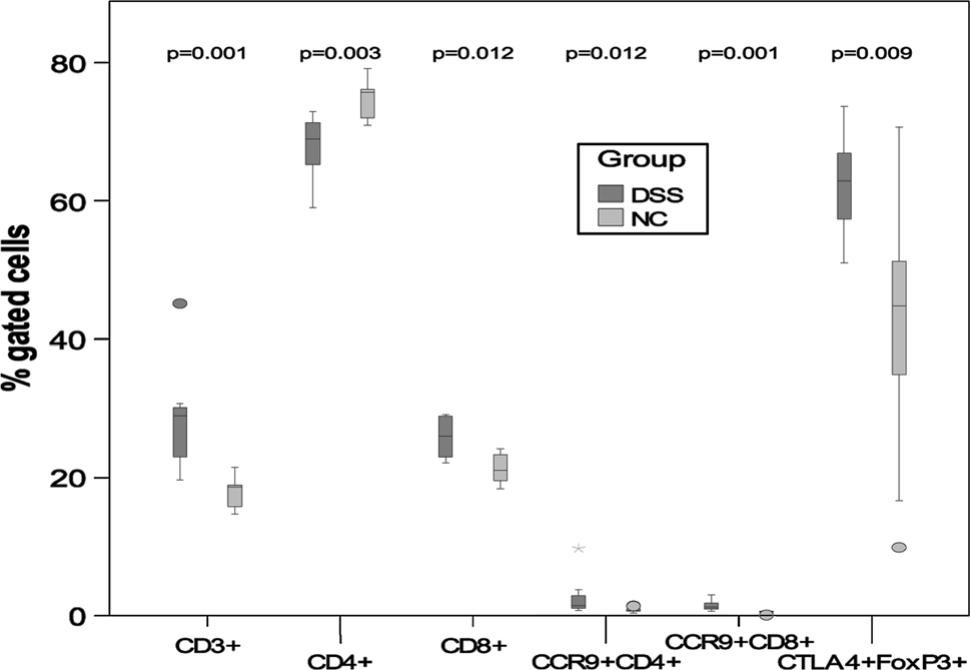
Statistical differences of percentage gated cells from Peyer′s patches. *P* values between the NC group and the DSS group are mentioned in the graph. Outliers are shown as *circle* and extreme values are shown as *asterisk*

Mononuclear cells were prepared from Peyer′s patches, mesenteric lymph nodes and spleen and analysed by FACS for the presence of various cell populations (Table 3). The percentage of CD3+ and CD8+ lymphocytes was significantly higher in Peyer′s patches isolated from the DSS group (*P* = 0.001 and *P* = 0.012, respectively), while the percentage of CD4+ was lower (*P* = 0.003) (Table 3; Fig 2). The percentages of the phenotype of CD4+ and CD8+ cells expressing the gut homing chemokine receptor CCR9 were significantly more numerous in the DSS group (*P* = 0.012 and *P* = 0.001), also were the percentage of regulatory T cells defined as CD4+CD25+ CTLA-4+FoxP3+ (*P* = 0.009) (Table 3; Fig. 2).

**Table 3:** Results of FACS analysis showing median with minimum and maximum values within parenthesis

Cells	Group	Mesenteric lymph nodes			Peyer’s patches			Spleen			
		n	Median (min–max)	*P* ^a^	n	Median (min–max)	*P* ^a^	n	Median (min–max)	*P* ^a^	*P* ^b^
T cells (CD3+), T-helper (CD4+), Cytotoxic T cells (CD8+) and B cells (B220+)											
CD3+	DSS	9	40.6 (35.4–62.7)		8	28.9 (19.7–45.1)		10	30.5 (24.6–43.5)		0.034*
	NC	10	59.2 (21.4–73.4)	0.060	9	18.6 (14.7–21.4)	0.001**	10	31.0 (28.8–35.7)	0.31	0.001**
CD4+	DSS	9	55.4 (51.9–57.5)		8	68.9 (59.0–72.8)		10	57.8 (55.9–62.7)		0.002**
	NC	10	56.9 (51.0–75.7)	0.19	9	75.7 (70.9–79.1)	0.003**	10	58.0 (54.6–65.2)	0.65	0.002**
CD8+	DSS	9	42.0 (38.9–45.0)		8	26.0 (22.1–29.2)		10	37.2 (33.0–40.0)		0.001**
	NC	10	40.1 (19.8–46.5)	0.22	9	21.0 (18.3–24.1)	0.012*	10	36.3 (28.7–39.4)	0.70	0.001**
B220+	DSS	9	54.5 (33.1–60.0)		8	66.1 (9.53–76.9)		10	63.2 (46.9–66.3)		0.20
	NC	10	36.3 (24.2–76.8)	0.12	9	79.2 (14.5–83.9)	0.054	10	60.7 (51.3–62.2)	0.16	0.099*
Recruited T cells (CCR9+CD4+/CD8+) and activated T cells (CD4+/CD8+CD69-/CD69+)											
CCR9+CD4+	DSS	9	1.22 (0.33–8.72)		8	1.43 (0.73–9.74)		10	0.70 (0.22–1.03)		0.008**
	NC	10	1.32 (0.34–6.28)	0.65	9	0.75 (0.34–1.44)	0.012*	10	0.44 (0.20–1.71)	0.45	0.011*
CD4+CD69-	DSS	9	49.0 (37.1–61.2)		8	30.6 (12.7–45.0)		10	63.2 (44.7–76.9)		0.002**
	NC	10	52.7 (30.6–80.6)	0.87	9	21.5 (15.7–80.6)	0.15	10	58.7 (26.3–74.5)	0.50	0.008**
CD4+CD69+	DSS	9	51.0 (34.3–62.9)		8	69.4 (55.0–87.3)		10	36.8 (23.1–55.3)		0.002**
	NC	10	47.3 (9.00–69.4)	0.94	9	77.3 (9.00–84.3)	0.39	10	39.7 (25.5–73.7)	0.65	0.008**
CCR9+CD8+	DSS	9	0.80 (0.32–2.31)		8	1.23 (0.71–3.00)		10	0.42 (0.18–1.48)		0.044*
	NC	10	0.78 (0.09–2.32)	0.62	9	0.51 (0.09–0.77)	0.001**	10	0.40 (0.15–1.80)	0.82	0.027*
CD8+CD69-	DSS	9	57.3 (44.4–80.6)		8	26.2 (5.88–69.4)		10	76.1 (66.6–84.9)		0.011*
	NC	10	61.8 (17.6–82.4)	0.68	9	37.6 (17.6–56.8)	0.50	10	78.2 (57.4–89.9)	0.60	0.001**
CD8+CD69+	DSS	9	40.2 (11.5–55.5)		8	72.2 (30.6–94.1)		10	21.7 (15.1–28.7)		0.002**
	NC	10	38.2 (17.6–82.4)	0.68	9	54.0 (37.9–82.4)	0.21	10	21.5 (10.1–42.6)	0.70	0.001**
Monocytes (CD11b+CD11c+), macrophages (CD11b+CD11c-) dentritics (CD11b-C11c+) and bacteria induced activation (CD11c+TLR4+)											
CDllb+CDllc+	DSS	9	22.9 (13.5–50.8)		8	12.0 (2.15–27.6)		10	11.2 (6.44–26.2)		0.002**
	NC	10	9.12 (1.01–18.9)	0.003**	9	11.3 (1.49–16.7)	0.77	10	10.8 (3.68–16.9)	0.60	0.64
CD1lb+CDllc-	DSS	9	10.78 (5.74–17.81)		–	–		–	–		–
	NC	9	8.59 (2.47–15.73)	0.31	–	–	–	–	–	–	–
CDllb-CDllc+	DSS	9	5.23 (2.12–16.75)		–	–		–	–		–
	NC	9	8.57 (4.65–15.25)	0.058	–	–	–	–	–	–	–
CDllc+TLR4+	DSS	9	14.2 (2.12–21.4)		8	5.57 (0.88–9.76)		10	18.5 (2.39–35.0)		0.030*
	NC	10	5.62 (0.30–16.4)	0.018**	9	3.44 (2.02–8.09)	0.63	10	23.2 (1.80–38.0)	0.88	0.045*
Intracellular (CD4+CD25+)											
CD4+CD25+FoxP3+	DSS	9	15.5 (14.3–18.4)		7	12.7(10.4–15.8)		10	17.6 (5.13–21.2)		0.028*
	NC	10	8.51 (4.52–16.1)	0.004**	9	11.6 (7.88–14.4)	0.10	10	16.4 (12.7–22.6)	0.23	0.004**
CD4+CD25+CD69+FoxP3+	DSS	9	37.6 (14.5^2.6)		8	36.5 (29.3–52.0)		10	29.1 (21.4–32.1)		0.010*
	NC	10	13.6 (3.29–45.6)	0.034*	9	39.0 (3.99–51.3)	0.92	10	27.3 (23.4–36.4)	0.50	0.032*
CD4+CD25+CTLA4+FoxP3+	DSS	9	70.0 (61.0–81.6)		8	62.8 (51.0–73.7)		10	73.1 (57.7–81.6)		0.010*
	NC	10	34.4 (7.84–76.1)	0.003**	9	44.7 (9.88–70.6)	0.009**	10	47.0 (25.8–78.7)	0.005	0.72
Regulatory T cells (CD4+/CD8+CD25+) and NK cells (CD4+/CD8+CD49b+)											
CD4+CD25+	DSS	9	17.2 (6.06–25.6)		8	9.34 (2.29–21.1)		10	16.7 (9.86–20.0)		0.093
	NC	10	13.9 (10.5–21.9)	0.22	9	11.0 (5.37–14.0)	0.21	10	15.3 (8.08–19.6)	0.15	0.004**
CD8+CD25+	DSS	9	3.10 (1.14–6.81)		8	6.04 (1.77–15.1)		10	2.24 (0.94–5.61)		0.072
	NC	10	2.20 (0.69–14.0)	0.68	9	4.41 (2.92–6.53)	0.44	10	2.64 (1.24–4.30)	0.36	0.032*
CD4+CD49b+	DSS	9	1.13 (0.79–2.10)		8	2.12 (1.10–.89)		10	4.46 (2.62–6.11)		0.011*
	NC	10	0.88 (0.45–1.98)	0.37	9	1.48 (0.92–2.18)	0.054	10	3.48 (1.48–5.71)	0.049	<0.001***
CD8+CD49b+	DSS	9	1.15 (0.55–1.35)		8	4.70 (2.19–17.3)		10	2.36 (0.92–3.08)		0.001**
	NC	10	1.05 (0.73–3.44)	0.87	9	2.93 (1.47–.97)	0.054	10	2.26 (0.34–3.27)	0.76	0.062
Number of cells	DSS	8	26.2 (13.9–69.0)		8	4.25 (1.00–7.00)		8	188 (37.5–371)		–
	NC	8	7.95 (3.30–23.0)	0.002**	8	10.6 (0.37–29.8)	0.021*	8	189 (44.0–333)	0.88	–

*P < 0.05, ** *P* < 0.01 and *** *P* < 0.001 between groups (P^a^) or within groups (P^b^)

^a^Comparison between groups by means of the Mann–Whitney U test

^b^Comparison between organs within each group by means of the Friedman’s test

**Fig. 3:**
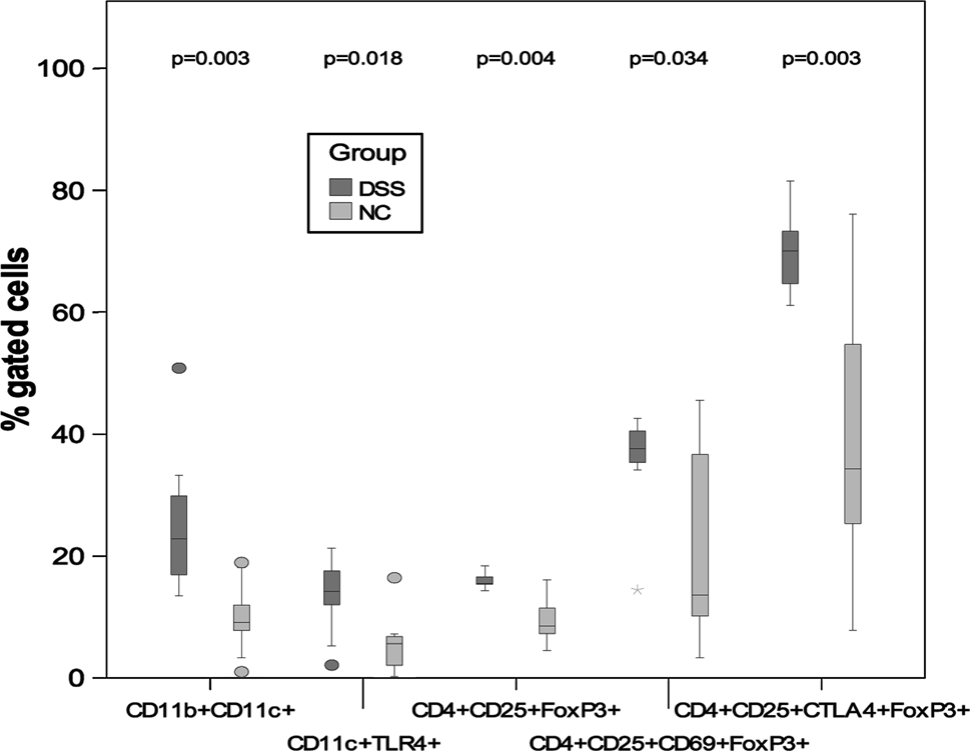
Statistical differences of percentage gated cells from mesenteric lymph nodes. *P* values between the NC group and the DSS group are mentioned in the graph. Outliers are shown as ° and extreme values are shown as *asterisk*

The percentage of double-positive CD11b and CD11c phagocytes and CD11c+TLR4+ dendritic cells was significantly increased in mesenteric lymph nodes from the DSS group (*P* = 0.003 and *P* = 0.018, respectively) (Table 3; Fig. 3). Also, the percentages of CD4+CD25+ regulatory T cells, expressing Foxp3 and CD69 or CTLA-4, were higher (*P* = 0.004, *P* = 0.034, *P* = 0.003) (Table 3; Fig. 3).

**Fig. 4:**
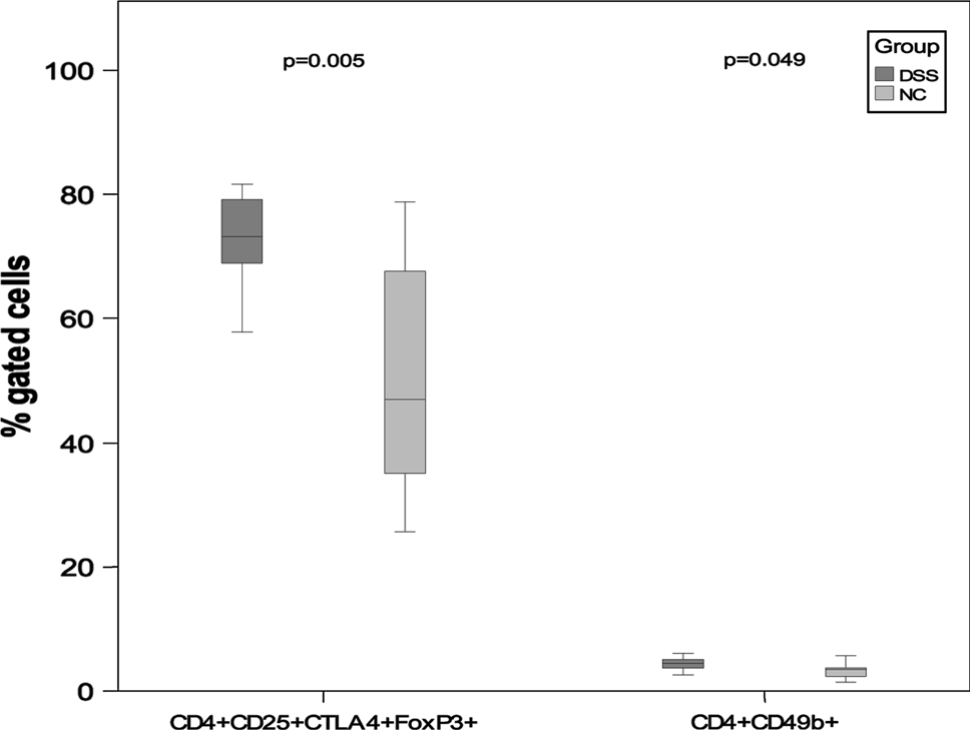
Statistical differences of percentage gated cells from spleen. *P* values between the NC group and the DSS group are mentioned in the graph. Outliers are shown as *circle* and extreme values are shown as *asterisk*

In the spleen, we found a significant increase in the frequency of regulatory T cells (CD4+CD25+ CTLA-4+FoxP3+) and CD4+CD49+ NK cells, in the DSS group (*P* = 0.005, *P* = 0.049) (Table 3; Fig. 4).

A significant increase in total cell number was found in mesenteric lymph nodes in the DSS group, whereas fewer cells were found in Peyer′s patches compared to the NC group (Table 3).

### Correlations

A linear relationship was observed between DAI at day 7 and the incidence of *Enterobacteriaceae* (*r* = 0.84; *P* < 0.001) as well as between the DAI score and colonic MPO activity (*r* = 0.70; *P* < 0.001). When correlating DAI to CD4+/CD25+/FoxP3 and CD4+/CD25+/CTLA-4+/FoxP3 cells, positive correlations were found (*r* = 0.54; *P* = 0.018 and *r* = 0.54; *P* = 0.017). Positive correlations were also found between CD11b+/CD11c+ cells and CD4+/CD25+/FoxP3+cells (*r* = 0.69; *P* < 0.001), CD4+/CD25+/CD69+/FoxP3+ cells (*r* = 0.67; *P* = 0.01) and CD4+/CD25+/CTLA-4+/FoxP3+ cells (*r* = 0.67, *P* = 0.01).

### Multiplex cytokine profiling

The levels of 11 cytokines/chemokines were measured in parallel following induction of colitis (Table 4). Acute DSS-induced colitis displayed a cytotoxic and chemotactic serum profile with significantly elevated levels of IL-6, IL-17 and KC (*P* = 0.008, *P* = 0.017 and *P* = 0.006, respectively) (Table 4).

**Table 4:** Concentrations (pg/ml) of cytokines/chemokines in serum

Serum					
Cytokine/chemokine	Group	n	Median	25–75 %	*P* value^a^
KC	NC	7	27.57	7.18–50.57	
	DSS	9	669.63	376.73–2244.24	0.006**
IL-6	NC	7	0.67	0.67–17.57	
	DSS	9	74.13	42.35–85.00	0.008**
IL-17	NC	7	0.91	0.54–1.32	
	DSS	9	3.05	1.87–460	0.017*
TNF-α	NC	7	0.81	0.81–2.03	
	DSS	9	3.54	0.057	
IL-5	NC	7	6.80	4.87–12.78	
	DSS	9	4.12	0.169	
IL-4	NC	7	0.93	0.73–0.98	
	DSS	9	0.73	0.220	
IL-12	NC	7	46.84	2.46–75.03	
	DSS	9	5.61	0315	
IL-2	NC	7	1.06	1.06–4.00	
	DSS	9	1.53	0.396	
IL-Iβ	NC	7	2.31	2.31–3.16	
	DSS	9	2.60	0.523	
IFN-γ	NC	7	17.12	0.81–27.59	
	DSS	9	3.51	0.711	
IL-10	NC	7	3.50	1.82–21.83	
	DSS	9	5.0	0.916	

** *P* < 0.01 and * *P* < 0.05 compared to NC group

^a^Mann-Whitney U test.

### Intestinal microbiota

Eleven T-RFs of different size were detected with significantly different occurrence between the two groups. Eight of the T-RFs were only detected in the DSS group (Table 5).

**Table 5:** Size of T-RFs detected in the samples from the two groups that have significantly different frequency of occurrence

T*-*RFs	NC	DSS	Significance
88.76	6	1	*P* = 0.029*
93.59	0	8	P < 0.001***
94.73	0	4	*P* = 0.043*
141.29	0	4	*P* = 0.043*
156.03	0	4	*P* = 0.043*
183.79	6	0	*P* = 0.005**
200.52	0	4	*P* = 0.043*
203.83	8	1	*P* = 0.003**
298.71	0	4	*P* = 0.043*
489.83	0	4	*P* = 0.043*
543.55	0	4	*P* = 0.043*

* *P* < 0.05, ** *P* < 0.01 and *** *P* < 0.001 between NC DSS groups

**Fig. 5:**
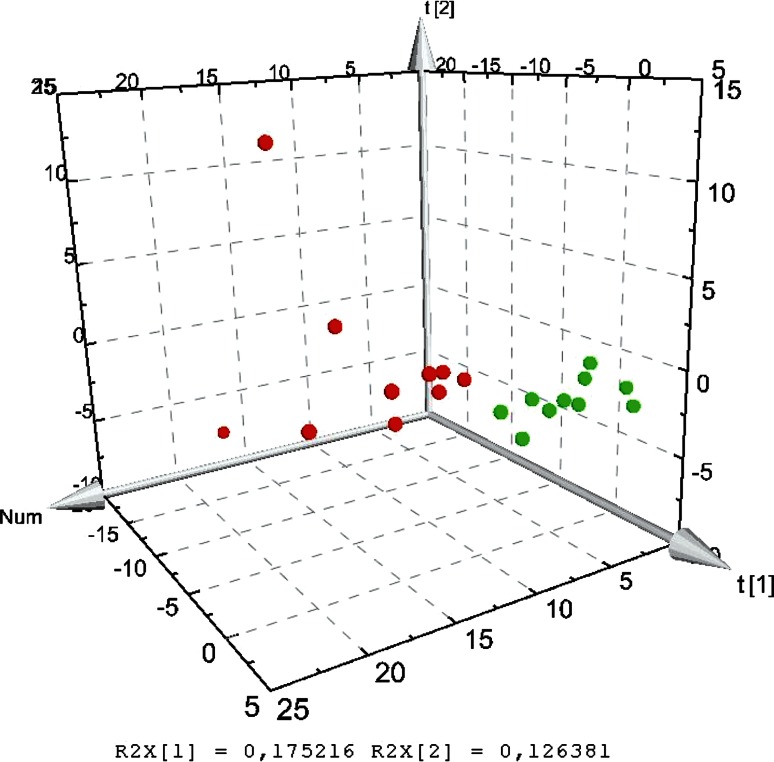
PCA 3D score scatter plot of T-RFLP data obtained from *Msp*I digestion

On the basis of the analysis of the mucosal bacterial communities by T-RFLP patterns, the microbial diversity was calculated using the peak area of each sample, expressed as the proportion of the total area for a sample. The median values from Shannon–Wiener diversity index (H’) were 2.52 (2.35–2.69) for the NC group and 2.55 (2.16–2.77) for the DSS group. The difference was not significant. PCA analysis of T-RFLPs obtained from each individual showed that the distribution of the microbial communities was clearly distinct between the groups (Fig. 5).

**Fig. 6:**
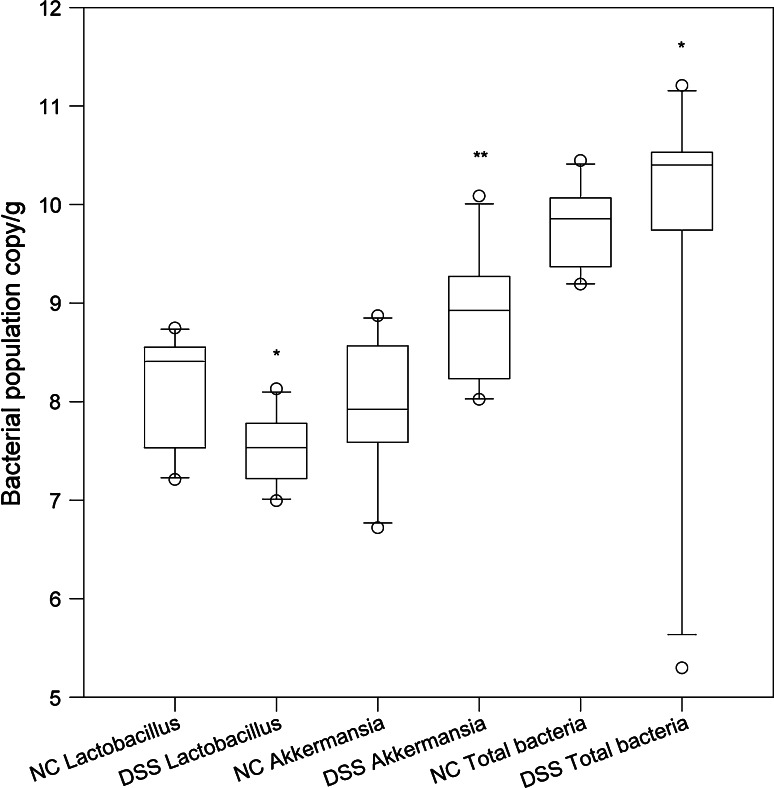
Comparison of bacterial populations determined by qPCR. *P < 0.05 and **P < 0.01 compared to the NC group

The quantification of total amount of bacteria on colonic mucosal samples showed significantly more bacteria in the DSS group 2.6 × 10^10^ copies of 16S rRNA genes/g compared to the NC group 7.6 × 10^9^ (*P* = 0.025) (Fig. 6). The amount of *Lactobacillus* was significantly lower in the DSS group, NC group 2.6 × 10^8^ copies/g and DSS group 3.4 × 10^7^ copies/g (*P* = 0.026) (Fig. 6), while the amount of *Akkermansia* and *Desulfovibrio* was significantly higher in the DSS group, NC group 8.6 × 10^7^ copies/g and DSS group 8.6 × 10^8^ copies/g (*P* = 0.005) and NC group 3.6 × 10^6^ copies/g and DSS group 1.4 × 10^7^ copies/g (*P* = 0.025), respectively (Fig. 6).

Only one animal in the NC group reached the detection range for *Enterobacteriaceae*, i.e. with the calculated value of 3.9 × 10^6^copies/g. The incidence of *Enterobacteriaceae* was significantly different between the groups. In the NC group, *Enterobacteriaceae* was found in 1 out of 10 animals compared to 9 out of 10 animals in the DSS group (*P* < 0.001).

### PCA of cellular findings and microbial communities

**Fig. 7:**
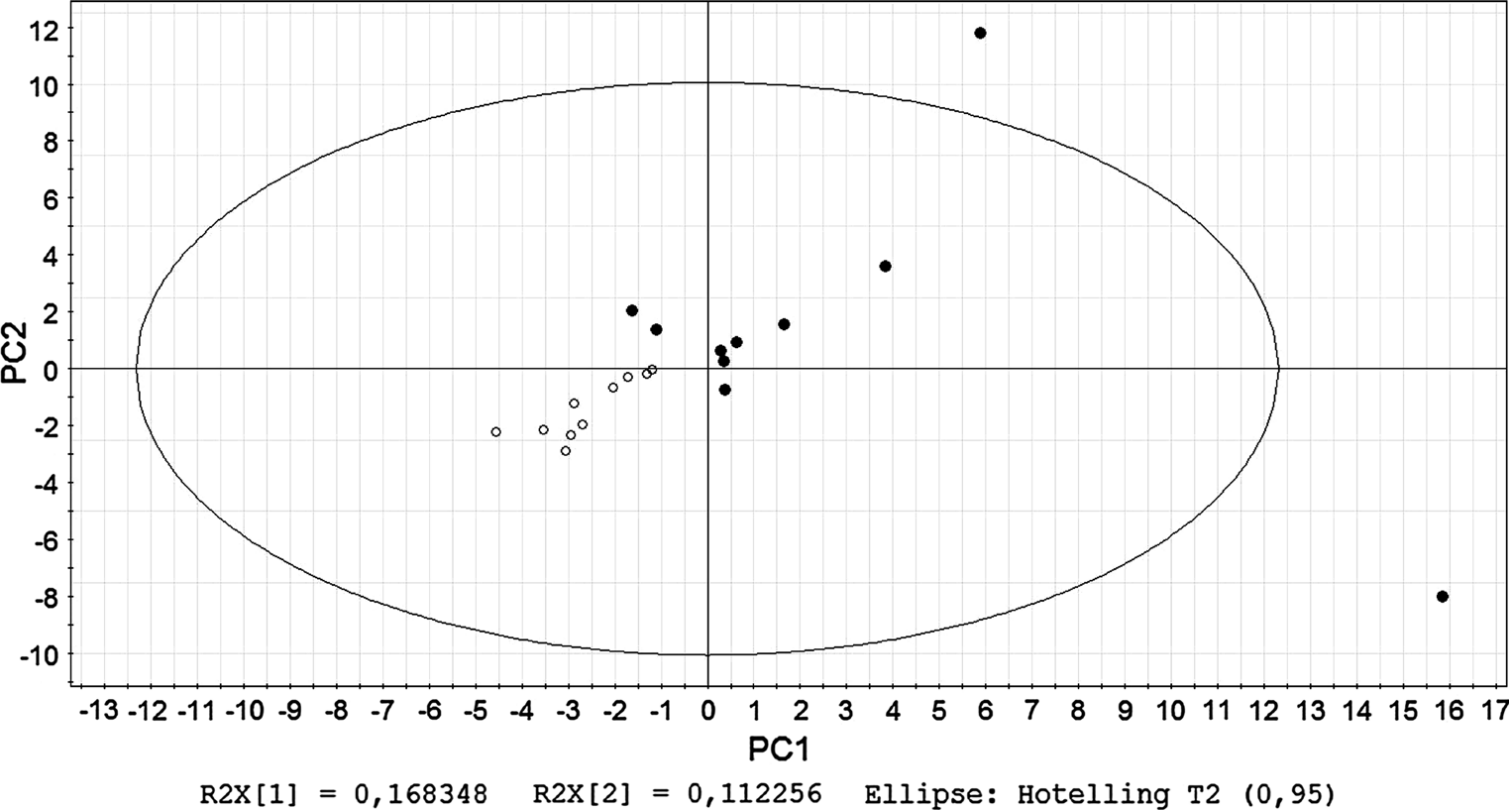
PCA score scatter plot of T-RFLP data obtained from *Msp*I digestion together with flow cytometry data measured from mesenteric lymph nodes. *filled circles* represent animals from the NC group, and *open circles* represent those from the DSS group

PCA models were built on both of the FACS data and T-RFLP data (Fig. 7). The NC and DSS groups were separated in all the models, while Peyer’s patches showed different expression pattern of the markers compared to lymph nodes and spleen. The microbiota was also altered after DSS treatment.

## Discussion

The DSS-induced colitis resembles UC due to the clinical symptoms, inflammatory markers and histopathological changes [7, 10]. Mice strains show differential susceptibilities to DSS, and the possibility of C57BL/6 mice to progress to chronicity offers a promising animal model for the pathological inflammatory changes observed in UC [10].

DSS was given for 7 days to induce acute intestinal inflammation verified by decreased body weight, loose faeces/diarrhoea and rectal bleeding, and a significant difference in DAI was found compared to the NC group (Table 2). Feed intake was significantly lower in the DSS group, and signs of oedema were visible in both caecum and colon, confirmed by tissue weights and length (Table 2). The clinical symptoms were associated with the presence of crypt distortion, erosion and inflammation (Fig. 1b, c), but no histopathological changes could be observed in the liver.

MPO is an enzyme found in neutrophils and in active UC patients, rectal MPO concentrations correlated with mucosal structural changes and the increase in inflammatory infiltrate [25]. The presence of neutrophils in the lamina propria and in the epithelium also correlated with the neutrophil marker MPO [25]. In the present study, MPO activity was significantly increased in colon (*P* < 0.001) but not in the small intestine, indicating colonic infiltration of neutrophils. A positive correlation between DAI scoring and colonic MPO activity was found (*P* < 0.001).

The global composition of the gut microbiota may be more relevant to the pathogenesis of UC than the presence of certain pathogens. By the use of T-FRLP, microbial community structures can be compared and microbial diversity assessed. T-RFs unique to the DSS group were found (Table 5), and PCA based on T-RFLP indicated distinct patterns of the microbiota between the two groups (Fig. 5), although in contrast to former presented data, the diversity of the microflora was not significantly different between the groups [26].

Detailed knowledge on the gut microbiota composition in acute intestinal inflammation is still limited, but certain genera such as *Bacteroides* and *Clostridium* have been implicated in the induction of inflammation, while a significant decrease in *Lactobacillus* is found during UC [27, 28]. A high proportion of pro-inflammatory species such as *Pseudomonas aeruginosa*, *Bacteroides fragilis* and *Clostridium difficile* was found dominating the microbiota on mucosa of a patient with acute UC, and the ileal pelvic pouch microbiota from two former ulcerative colitis patients showed the presence of *Clostridium perfringens* [29, 30]. To determine whether certain members of the microbiota or the microbial mass are responsible for the abnormal microbe–immune system interplay is not a simple process. Significantly higher 16S rRNA gene copy numbers of all mucosal bacteria were found after DSS administration (Fig. 6), which is in agreement with the findings of Bibiloni et al. where UC patients had higher numbers of bacteria associated with biopsies than healthy subjects [31]. Janeczko et al. [32] also demonstrated that the total number of mucosal bacteria was strongly associated with changes in mucosal architecture and the density of cellular infiltrates, particularly macrophages and T cells. It is speculated that the difference may reflect the altered mucus present on the mucosal surface of colons from UC patients, which is thinner and less sulphated than normal [33, 34]. A thin mucus layer, containing larger numbers of bacteria than normal, might facilitate contact between bacterial antigens and the mucosal immune system [31]. Increased numbers of intestinal sulphate-reducing bacteria, such as those of the genus *Desulfovibrio,* and rates of sulphidogenesis have been associated with UC and correlated with reduced mucosal thickness [35–37]. In accordance with these findings, we found significantly higher numbers of the 16S rRNA gene copies in colonic mucosa in the DSS group (Fig. 6). *Akkermansia* *muciniphila* is a mucin-degrading Gram-negative bacterium isolated from human mucosa [38], and by quantification of the gene copies, significantly higher numbers were obtained in the same group (Fig. 6). However, it is not known if *Akkermansia* *muciniphila* has any sulphate-reducing capacity. The genera *Lactobacillus* can be considered as beneficial during DSS-induced acute colitis [14, 39]. In the present study, the amount of lactobacilli on colonic mucosa was suppressed (Fig. 6). Conversely, members of the epithelium-associated *Enterobacteriaceae* (*Eschericha coli*) counts have been shown to be higher during active UC, and the incidence of mucosal *Enterobacteriaceae* was higher in the DSS group [40]. An interrelationship in the form of a positive correlation between *Enterobacteriaceae* and DAI was found, which has also been verified during feline inflammatory bowel disease [32].

Intestinal macrophages play an important role in mucosal inflammation. In contrast to normal mucosa, there is a significantly higher expression of CD11b and CD11c in colonic macrophages during UC [41]. Flow cytometric analysis was applied to study CD11b and CD11c double-positive cells from spleen, Peyer′s patches (PP) and mesenteric lymph nodes (MLN), and a significantly increased population of these cells were found in MLNs from the DSS group (Table 3; Fig. 3). It has been hypothesized that this may reflect either a recruitment of new cells from the circulation or a change in phenotype of resident cells [41]. Determination of total cell number confirmed that there were significantly more cells in MLN of the DSS group than in the NC group, whereas fewer cells were found in PP (Table 3).

Lipopolysaccharide (LPS) derived from Gram-negative bacteria is a major inducer of inflammatory responses. TLR4 is involved in LPS signalling and serves as a cell-surface co-receptor for CD14, leading to LPS-mediated NF-kB activation and subsequent cellular events. TLR4 is expressed in cells that respond to LPS, such as peripheral blood leucocytes, monocytes, macrophages and dendritic cells [42]. An increase in TLR-4 mRNA in inflamed colonic tissue [43] as well as in TLR-4-positive intestinal DC confined to areas of inflamed tissue in UC patients has been observed [44]. Here, we found an increased proportion of cells expressing CD11c and TLR-4 in MLN during colitis induction (Table 3; Fig. 3), which is consistent with the increasing amounts of *Enterobacteriaceae* and *Akkermansia* found on colonic mucosa. Increased expression of TLRs by DCs and other cells interacting with the microbiota may lead to increased recognition and enhanced, or changed, responses due to an abnormal bacterial composition [44]. TLR ligands have been suggested to promote the recruitment and/or proliferation of regulatory T cells, since FoxP3-positive cells were significantly decreased in DSS-treated TLR2-/-, TLR4-/- and TLR2/4-/- mice [45]. This may be consistent with similar observations in UC, where an increased frequency of CD4+CD25+FoxP3+ T cells were found in mucosal lymphoid tissue during UC [46]. In some cases, a dense population of FoxP3+ T cells has been reported to be found in T-/B cell boundaries, indicating the potential interaction of these cells with antigen-bearing DC [46]. In the present study, the percentage of CD4+CD25+FoxP3+ expressing T cells in MLN was increased (Table 3, Fig. 3). Increased percentages of CD4+CD25+FoxP3+ cells co-expressing CD69 and CTLA-4 were also shown (Table 3; Fig. 5) as well as positive correlations between CD11b+/CD11c+ cells and CD4+CD25+FoxP3+ cells with or without co-expression of CD69 (T-cell activation marker) and CTLA-4 (constitutively expressed by regulatory T cells). Why the CD4+CD25+FoxP3+ regulatory T cells, despite increased frequency, fail to control the development of colitis has been a forum for speculations [46]. The suppressive activity may be abrogated through either co-stimulatory molecules or TLR signalling. It could also be that the high numbers of cells to some extent limit the severity of inflammation in the affected colon, despite their inability to reverse the disease process [46] or that strong T-cell receptor stimuli may abrogate the suppressive activity or render the effector T cells resistant to suppression [46]. IL-6 secreted from splenic DCs upon TLR stimulation has been shown to be an important factor of T-cell activation by overcoming regulatory T-cell-mediated suppression of T-cell proliferation [47]. The serum cytokine profile in the present study was characterized by IL-6, IL-17 and the chemokine KC (Table 4). These findings are in accordance with a previous study by Alex et al. [48]. The Th17 pathway that is mediated by IL-17 and IL-23, which is essential for the manifestation of chronic intestinal inflammation [49] and in colonic specimens, the IL-17 gene expression in UC was increased in moderate to severe disease [50]. No structural homologue of IL-8 has been identified in mice, but KC shares many functional properties with human IL-8 and is capable of recruiting neutrophils [51].

Although colon is known to be the primary site of DSS action, the small intestine (jejunum–ileum) has been reported to be affected, albeit to a minor extent [11]. However, this could not be verified by histology in the present study. On the other hand, the segments were taken from duodenum–jejunum, and the affected areas may be more prominent in ileum. Cellular phenotyping by flow cytometry of PP and spleen demonstrated an increased proportions of CD3+, CD4+ and CD8+ T cells as well as CD4+CD25+FoxP3+ cells, co-expressing CTLA-4 in the DSS group (Table 3; Fig. 2). The only difference found in spleen was the increased percentage of CD4+CD25+FoxP3+ cells, co-expressing CTLA-4 and CD4+CD49b+ cells (Table 3; Fig. 4). In PP, the percentage of CD4+ and CD8+ cells, co-expressing the chemokine receptor CCR9, was also increased in the DSS group (Table 3; Fig. 2). These results are in line with previous findings in inflamed gut [52] and confirm an involvement of the small bowel in the disease process. The number of cells in PP in the present study did not correlate with the numbers of patches found, and all PP found in each animal were collected to determine the absolute cell number. Since the total cell number in PP decreased after DSS administration, it is difficult to determine whether the observed increase in the percentage of different cell types is due to increased infiltration of specific cells and to the loss of other cell types or if it is because of a relative enrichment due to general cell depletion consequent of the inflammatory status. In the absence of MLNs, it is known that an intestinal inflammation in mice becomes more severe, whereas the absence of PPs alone does not affect disease severity [53]. Based on these observations, a regulatory function for MLNs has been suggested, but not for PPs in intestinal inflammation [53].

In the present study, principal component analysis was used to evaluate the complex relationship between multiple immune parameters and the composition of the colonic microbiota. When T-RFs were combined with cellular findings from the flow cytometry analysis, distinct differences between the groups were appeared (Fig. 7), suggesting a possible role for the gut microbiota in the pathogenesis of experimental colitis by affecting immunological responses in the gut.

In conclusion, DSS-induced colitis in C57BL/6 mice show similarity in appearance to UC in many ways, and our results indicate that intestinal bacteria actively interact with the immune system. The results highlight the importance to evaluate the composition of the microbiota during colonic inflammation. Further studies should address the correlation between quantitative changes in enteric microbial composition and immunological parameters to clarify the pathogenesis of UC and to determine whether the use of specific bacteria may be helpful to treat colitis in humans.
